# The effect of gender-specific labor market conditions on children’s weight

**DOI:** 10.1186/s13561-021-00345-9

**Published:** 2021-12-02

**Authors:** Bongkyun Kim, Michael R. Thomsen, Rodolfo M. Nayga, Anthony Goudie

**Affiliations:** 1 Department of Eonomics, Kangwon National University, 1 Gangwondaehakgil, Chuncheon-si, Gangwondo Republic of Korea 25913; 2 Fay W. Boozman College of Public Health, University of Arkansas for Medical Sciences, 4301 W. Markham, Slot 820, Little Rock, AR 72205 USA; 3 Department of Agricultural Economics, Texas A&M University, 600 John Kimbrough Blvd, Suite 309, College Station, TX 77843 USA; 4 Fay W. Boozman College of Public Health, University of Arkansas for Medical Sciences, Director of Research and Evaluation Arkansas Center for Health Improvement, 1401 West Capitol Suite 300, Little Rock, AR 72201 USA

**Keywords:** Labor market conditions, Child overweight, Preparing home-cooked foods, Time use data

## Abstract

**Background:**

Macroeconomic conditions are widely known to influence health outcomes through direct behavioral change or indirect mental effects of individuals. However, they have not received much attention in relation to childhood obesity.

**Methods:**

Using gender-specific predicted employment growth rates as an index for labor market conditions, we analyze how economic shocks affect children’s weight status in Arkansas. To understand the underlying mechanisms behind these results, we use data on individual time use to examine how economic shocks are related to activities related to children’s weight.

**Results:**

Improvement in the female labor market is associated with an increase in body mass index (BMI) and the probability that a child is overweight or obese, while an improvement in the male labor market has no significant effects on children’s weight. This impact is particularly evident among female children, older children, and African-American children. We also find a negative effect of improvements in the female labor market on time spent on preparation for foods at home.

**Conclusions:**

These results suggest that a decrease in time spent preparing home-cooked foods might be a plausible explanation for the pro-cyclical relationship between children’s weight and improvement in the labor market conditions. Thus, the policy implications of our paper should be aimed at mitigating the adverse effects of women’s labor participation.

## Introduction

Obesity during childhood is highly correlated with adverse health and development outcomes [1–6]. As obesity rates for children between 2 and 19 years of age have increased over the past decades in the United States [7], economists have studied the factors that may contribute to childhood obesity, including changes in the home, school, and food environments [8–12]. Macroeconomic conditions are widely known to influence health outcomes. There is a body of literature showing significant correlations between economic fluctuations and a variety of other health outcomes among adults, such as mortality, drinking, smoking, mental health, and body mass index (BMI) [13–19]. Nevertheless, economic shocks have received limited attention in relation to childhood obesity.

There are several potential mechanisms whereby overall economic conditions can affect children’s weight status [20]. For instance, a decrease in household income during a recession can lead to a reduction in parental health investments. Parental stresses from job insecurity can, in turn, increase children’s stress level, which may lead to poor dietary behaviors. On the other hand, because the opportunity costs of spending time with children may be lower during economic downturns, parents may spend more time caring for children in the home. Furthermore, economic conditions facing males and females may differently influence children’s weight [21]. Economic theory on division of labor in households [22] and data from the American Time Use Survey (ATUS) presented later in this paper suggests that males devote more of their time to the labor market, while females are more engaged with children. To the extent that men provide a majority of household income [23], the change in economic conditions that males face may have a larger impact on children’s weight status through investments in health or food quality than a change in economic conditions for females. In contrast, because women spend more time than men with children and female labor supply is more sensitive to wage change [24], economic shocks for females could have a larger impact on children’s weight by substituting time-intensive childcare with market-based childcare (e.g., fewer meals prepared in the home and more fast-food meals). In fact, there is a growing literature showing that economic conditions facing males and females have different effects on children’s well-being [21, 25, 26]. These conflicting theoretical predictions suggest that the effect of macroeconomic shocks on children’s weight is an empirical issue.

In this paper, we analyze the effect of local economic conditions on children’s weight in Arkansas, a state with one of the highest rates of childhood obesity. In particular, following Page et al. [21] and Lindo et al. [26], we construct county-level predicted employment growth rates for males and females as an index for local economic conditions. This index allows us to isolate demand-induced change in labor market opportunities, which is plausibly exogenous in explaining the relationship between economic fluctuations and children’s weight. We find that overall predicted employment growth rate is not significantly related to children’s weight status. However, when we consider gender-specific effects, an increase in the predicted female employment growth rate is significantly associated with an increase in BMI and the probability of a child being overweight or obese. In particular, the results indicate that a one percentage point increase in the predicted female employment growth rate is related to a 0.5 percentage point increase in the probability of children being overweight or obese. This result is approximately equivalent to 2 ~ 3 h more work per week, which translates into about a 3% increase in the probability of an unhealthy weight status. When examining whether there is heterogeneity across sub-samples, we find that the impact is particularly evident among female children, children in higher school grades (older children), and African-American children, who are the largest minority group in Arkansas. In contrast, an increase in the predicted male employment growth rate is negatively associated with children’s weight, but the effects are not significant. Lastly, when looking at the underlying mechanism of these results using data on individual time use, we find evidence that there is a significantly large and negative relationship between improvement in the female labor market and time spent on preparation of foods at home. We also find a significantly negative effect of improvement in the female labor market on time spent on purchasing prepared food, but the magnitude of the effect is trivial compared with the magnitude of the decrease in the time spent on preparation of foods at home. These results suggest that a decrease in time preparing home-cooked foods may explain the pro-cyclical relationship between children’s weight and female labor market conditions.

This paper contributes to the literature by using a unique longitudinal administrative dataset to provide evidence on children’s health. Pointing out the inconsistency between studies using individual level data (e.g., individual job loss) and studies using aggregate-level data (e.g., unemployment on state-level), Currie et al. [27] identify the need for more research into the effect of economic conditions on health using individual-level longitudinal data. To that end, our study takes advantage of a longitudinal dataset covering the population of public schoolchildren from the state of Arkansas. These data cover the period from 2004 to 2015 and provide information on individual’s weight status and socioeconomic status. We are able to control for individual’s time-invariant characteristics that might affect both weight and local economic conditions where students reside. Second, we focus on aggregate-level economic conditions as a main factor that could change children’s weight status. A number of studies have investigated the relationship between the change in individual-level employment and children’s weight [28–38], but studies looking at the impact of macroeconomic fluctuation on children’s weight are relatively rare. As Ananat, Gassman-Pines, Francis, and Gibson-Davis [39] pointed out, the effect of community wide economic conditions differs from the effects of individual job status in that community-level economic conditions can capture additional effects beyond parental employment status. For example, during an economic boom, parents might spend less time caring for their children when they have a new job. However, even if parents are still unemployed, they may reduce time invested in childcare while looking for a new job. On the other hand, during an economic downturn, parents could experience psychological stress due to concerns about unemployment or gloomy outlook in the area, even if they do not experience a job loss. In other words, children’s weight status can be affected by economic conditions, regardless of parents’ employment status. Thus, an analysis of macroeconomic fluctuations could be more comprehensive than individual-level employment analysis given the variety of factors affected by the macro economy. Arkes [40] used the National Longitudinal Survey of Youth from 1997 (NLSY-97) to examine the relationship between state unemployment rates and adolescent weight and found that the weight of male adolescents is positively associated, whereas weight of female teenagers is negatively associated with economic conditions. Cotti and Simon [41] and Page et al. [21] exploit fluctuations in stock market and demand-induced labor market opportunities, respectively, to study the impact of changes in economic conditions on children’s health status. These studies show that children’s mental and physical health are worse during economic downturns. Compared to Arkes [40], our study focuses on a broader range of children starting from kindergarten, uses more recent data, examines labor-market conditions facing males and females separately, and uses individual fixed-effects to control for time-invariant factors within children. Moreover, we add to the studies of Cotti and Simon [41] and Page et al. [21] by focusing on a different health outcome, children’s weight status. Lastly, together with Anderberg et al. [25], Lindo et al. [26], and Page et al. [21], our paper is also one of the few studies examining the effect of gender-specific economic condition on children’s well-being.

## Methods

### Data

We use four datasets. The first two datasets are the Basic Monthly Current Population Survey (CPS) and 1990 Census of Population Social and Economic Characteristics. These datasets provide information on employment growth rates, which serve as an index for local economic conditions. The third dataset contains information on children’s body mass index (BMI) from the Arkansas’s legislatively mandated BMI screening program. Finally, the American Time Use Survey (ATUS) is used to explore the underlying mechanisms between economic conditions and children’s weight status.

Unemployment rates from Local Area Unemployment Statistics (LAUS) of the Bureau of Labor Statistics (BLS) and from the American Community Survey (ACS) are commonly used indicators for local economic conditions. However, these data have limitations in our context. First, the BLS’s LAUS series does not provide county-level unemployment rates by gender. While data for county-level unemployment rates by gender can be obtained from the ACS, the data exist for only 11 Arkansas counties (out of 75 counties) from the year 2005. Moreover, labor force, the denominator of the unemployment rate, varies with the labor supply. Thus, changes in unemployment rates may be related to changes in unobserved factors affecting children’s health, which biases results downward as explained by Page et al. [21].^1^ Following Page et al. [21] and Lindo et al. [26], we use eq. (1) to calculate predicted gender-specific employment growth rates for Arkansas counties to address these issues.

1 $$ {D}_{ctg}={\sum}_i{G}_{it}\ast \frac{E_{icg0}}{E_{cg0}} $$

where G_it_ is the statewide employment growth rate of industry *i* in year *t*, and E_icg0_/E_cg0_ is the gender-specific (g ∈ {female, male}) ratio of industry *i* employment in county *c* to total employment in county *c* during the 1990 base period (period 0). Industries used in constructing the index are “Agriculture, forestry and fisheries,” “Mining,” “Construction,” “Manufacturing,” “Wholesale and retail trade,” “Transportation and utilities,” “Information,” “Finance, insurance and real estate,” “Services,” and “Public administration”. Industry-specific data for the statewide employment growth rate come from the Current Population Survey [42]. Gender-specific industry ratios in each county during the base period come from the 1990 Census. We construct gender-specific and overall predicted employment growth rates for all counties from 2004 to 2013 based on 2002 and 2007 North American Industry Classification System (NAICS) codes. This index is often called a Bartik [43] or “shift-share” instrument, which provides a way of predicting variation in the variable of interest by combining local economic compositions with shifts at the aggregate level [44]. Since the variation in predicted employment growth rates is taken from statewide employment growth rates, there is an additional advantage of mitigating issues related to endogenous local labor supply shocks, a problem that occurs when using unemployment rates. However, for the Bartik or shift-share instrument to be valid, the local industry compositions need to be exogenous. Hence, in our context, the identification assumption is that the gender-specific industry composition of employment during the base period of eq. (1) is uncorrelated with local factors affecting children’s weight status.^2^ If this condition does not hold, the estimated effects will be biased. County fixed effects can control for county specific time-invariant factors, such as geographic features, but we acknowledge that other unobserved time-variant factors can be correlated both with industry composition of employment during the base period and children’s weight status. Therefore, the results should be interpreted with this potential bias in mind.

**Fig. 1:**
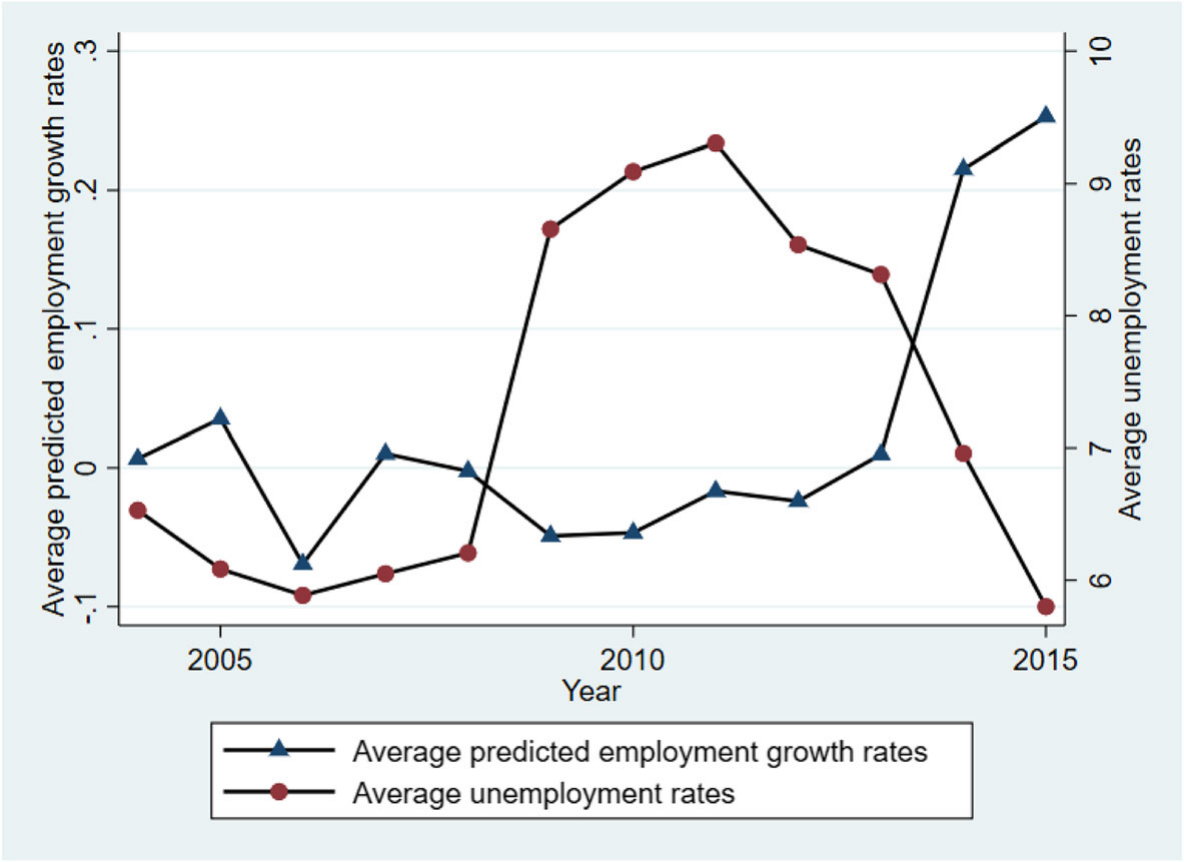
The trend of predicted employment growth rates and unemployment rates from BLS

Figure I presents trends of the average of the predicted employment growth rates, along with the employment rates from BLS. Overall, when the unemployment rates decrease, the predicted employment growth rates increase, indicating that they have opposite trends. We could not compare the trend by male and female, separately, because data by gender is unavailable from the BLS data. However, given the reverse relationship reflected in Fig. 1, we believe that there is likely to be similar patterns if this comparison were to be made by gender.

The childhood BMI dataset covers the 2003/2004 through 2014/2015 school years (hereafter 2004–2015). These data were collected through Arkansas’ statewide BMI screening program. During the in-school assessments, a school nurse conducted height and weight measurements while another individual served as the recorder. Measurements used a stadiometer, stabilized against a wall, and a digital scale. Prior to beginning the assessments, the scale was checked for accuracy. Children were asked to remove shoes, outerwear, glasses, jewelry and empty their pockets. Height was measured first. Weight was measured next and recorded to the nearest 0.1 kg. Finally, a second height measurement was taken. During the measurements the recorder verbally verified entry of the height data. If there was a disagreement between the two height measures that exceeded one inch, the measurement protocol was repeated; otherwise, the height used in BMI calculation was the average of the two measurements. A complete description of protocols and equipment used to assess children’s heights and weights is in the Arkansas Center for Health Improvement (ACHI) training document for public schools [45]. Schoolchildren are measured in kindergarten, 2nd, 4th, 6th, 8th, and 10th grades. Over the last 10 years, between 97.5 and 99.4% of Arkansas public schools participated in the program. These schools covered between 97.0 and 98.5% of public schoolchildren in these grades. Along with information on BMI z-scores and BMI percentiles, these data include information on children’s demographic characteristics, and whether the child qualifies for free or reduced-price school meals. BMI measures were converted to age and sex-specific z-scores according to the Centers for Disease Control and Prevention (CDC) guidelines [46]. Weight status measures were also based on the child’s BMI relative to the CDC reference growth charts. Those with BMIs at the 85th percentile and below the 95th percentile were classified as overweight.

ATUS provides information on how individuals spend time on a variety of activities over a 24-h period. Survey respondents report where and with whom each activity occurred and whether the activities were employment or business related. The ATUS also provides demographic information about the respondent, including sex, race, age, educational attainment, marital status, and the presence of children in the household [47]. We use data from the ATUS during 2006 ~ 2015, matching the period covered in our main analysis. Even though the ATUS is a helpful dataset to explore the mechanisms between labor market conditions and children’s weight, it has some limitations. Only one person whose age is at least 15 years old from each household is randomly selected for the survey. Moreover, because Arkansas is a relatively small state in terms of population, there are not many respondents from Arkansas in the ATUS. Hence, to ensure sufficient power for valid statistical inference in the analysis, we use data from all states in the ATUS.^3^

Summary statistics by data source are shown in Table 1. On average, the predicted female employment growth rate is almost identical to the predicted rate for males. Approximately 17 and 21% of children in our study are classified as overweight and obese, respectively. The majority of children are white, and over half of children are eligible for free or reduced-price meals. In the ATUS sample, there is a larger proportion of female respondents relative to male respondents. White is the majority race in the ATUS, and a majority of respondents earned a bachelor’s degree or higher and are full-time workers. Lastly, although not shown in Table 1, when looking at the time use for males and females separately, we find that females spend more time on activities that can affect children’s weight than do males.^4^

**Table 1:** Summary Statistics

	(1)	(2)	(3)	(4)
	Mean	Minimum	Maximum	Observations
**Current Population Survey (CPS) and 1990 Census**				
Predicted employment growth rate (female)^c^	1.3577 (4.2848)	−3.5331	14.8789	1,794,341
Predicted employment growth rate (male)^c^	1.3232 (5.6010)	−5.3915	17.1153	1,794,341
**Arkansas BMI panel**				
BMI z-score^c^	0.6913 (1.0743)	−3.9988	3.9996	1,794,341
Overweight^a^	0.1742 (0.3793)	0	1	1,794,341
Obesity^a^	0.2113 (0.4082)	0	1	1,794,341
Male^a^	0.5146 (0.4998)	0	1	1,794,341
Female^a^	0.4854 (0.4998)	0	1	1,794,341
Age^c^	10.2095 (3.4492)	4	18	1,794,341
White^a^	0.6605 (0.4735)	0	1	1,794,483
Hispanic^a^	0.0925 (0.2897)	0	1	1,794,483
Asian^a^	0.0185 (0.1346)	0	1	1,794,483
Black^a^	0.2188 (0.4135)	0	1	1,794,483
Others^a^	0.0097 (0.0982)	0	1	1,794,483
Free or reduced meal^a^	0.5708 (0.4950)	0	1	1,794,341
Full meal^a^	0.4292 (0.4950)	0	1	1,794,341
**American Time Use Survey (ATUS) data**				
Predicted employment growth rate (female)^c^	−0.4226 (0.3395)	−1.1365	0.5901	84,072
Predicted employment growth rate (male)^c^	− 0.7023 (0.4882)	−3.6830	0.9694	84,072
Food preparation^c^	37.9846 (57.8470)	0	995	84,072
Purchasing prepared food^c^	1.2212 (4.5570)	0	205	84,072
Physical activities with children^c^	8.5504 (38.6290)	0	840	84,072
Paid childcare and medical care^c^	0.5741 (9.8954)	0	1045	84,072
Male^a^	0.4327 (0.4955)	0	1	84,072
Female^a^	0.5673 (0.4955)	0	1	84,072
Age^c^	49.7287 (16.4049)	16	85	84,072
White^a^	0.8014 (0.3989)	0	1	84,072
African-American^a^	0.1435 (0.3506)	0	1	84,072
Others^a^	0.0550 (0.2281)	0	1	84,072
Less than high school^a^	0.1101 (0.3130)	0	1	84,072
High school^a^	0.2673 (0.4426)	0	1	108,434
Bachelor or equivalent degree^a^	0.4938 (0.5000)	0	1	108,434
Graduate degree^a^	0.1288 (0.3350)	0	1	108,434
Married with spouse^a^	0.5127 (0.4998)	0	1	108,434
Respondent working full time^a^	0.5112 (0.4999)	0	1	108,434
Spouse working full time^a^	0.3012 (0.4588)	0	1	108,434
Presence of own household children aged less than 18^a^	0.3829 (0.4861)	0	1	108,434
Number of household children aged less than 18^b^	0.7967 (1.1122)	0	10	108,434

Note: Mean values are represented. Standard deviations are in parentheses. a-Indicator variable, b-Counts, c-Units

### Empirical strategy

We first estimate eq. (2) using an individual fixed effects model to analyze the effect of general, not gender-specific, labor market conditions on children’s weight:

2 $$ {Y}_{jct}={\alpha}_0+{\alpha}_1{E}_{jct-2}+{\varphi}_i+{\delta}_c+{\gamma}_t+{\varepsilon}_{jct} $$

where *j* indicates the child. The *c* and *t* indexes are as defined in eq. (1). For *Y*, we use three outcomes: BMI z-score, an indicator for overweight or obesity, and an indicator only for obesity. The latter two indicators are defined in terms of the BMI percentile. Children with a BMI greater than the 85th and 95th percentiles are classified as overweight and obese, respectively. *E* indicates the overall predicted employment growth rate from eq. (1). That is ${E}_{jc}={\sum}_i{G}_{it}\times \frac{E_{ic0}}{E_{c0}}$. Since the overall employment growth rate is calculated over both males and females, there is no gender subscript for this index. In eq. (2), α_1_ indicates effect of a one percentage point increase in the overall predicted employment growth rate on children’s weight status. All observed and unobserved time-invariant characteristics of the child are controlled for by the individual fixed effects, φ_i_. County and year fixed effects are represented by δ_c_ and γ_t_, respectively. Following Lindo [48], robust standard errors are clustered by county and year to account for the possibility that the errors are correlated within areas over time and across areas within a given year. Lastly, we only include observations with a BMI z-score between ±4 because outside these ranges are values that are biologically implausible.

We next modify eq. (2) to model gender-specific growth rates. The predicted employment growth rate for males is defined as ${ME}_{jc}={\sum}_i{G}_{it}\times \frac{E_{icm0}}{E_{cm0}}$, and the predicted employment growth rate for females is defined as ${FME}_{jc}={\sum}_i{G}_{it}\times \frac{E_{icf0}}{E_{cf0}}$ from eq. (1). We use ME_ct_ and FME_ct_, instead of D_ctm_ and D_ctf_ to avoid confusion in notation for labor market conditions indexes specified in eq. (1). Following Page et al. [21], we include both male and female indices in the model. That is,

3 $$ {Y}_{jct}={\alpha}_0+{\alpha}_1{ME}_{jct-2}+{\alpha}_2F{ME}_{jct-2}+{\varphi}_i+{\delta}_c+{\gamma}_t+{\varepsilon}_{jct} $$

Thus, α_1_ (α_2_) represents the effect of a one percentage point increase in the predicted male (female) employment growth rate conditional on a constant female (male) employment growth rate. Because a change in weight status could take time to be observed following a change in behavior, we use a two-year lag for the predicted employment growth rates. Note that given the fact that predicted employment growth rates are closely related to unemployment rates and they are assumed to be exogenous changes in labor market opportunities, one could consider using the predicted employment growth rates as instrumental variables for unemployment rates. However, instrumental variable regression is not available because our variables of interest are labor market opportunities by gender, and there are no data on county-level unemployment rates by gender.

## Results

### The effect of labor market conditions on children’s weight

Table 2 presents estimates from eqs. (2)–(3). Results from eq. (2) are in the first row. The second and third rows contain results from eq. (3). In columns (1)–(3), we also present results for the BMI z-score, probability of being overweight or obese, and probability of being obese, respectively. The coefficients for the overall predicted employment growth rates in the first row are positive, but none are statistically significant. Thus, we can infer that overall labor market conditions do not statistically affect children’s weight status. However, when looking at the effect of the female labor market in the second row, we find that a one percentage point increase in the predicted female employment growth rate is significantly and positively associated with BMI, likelihood of being overweight or obese, and likelihood of being obese. The effect size is 0.0124 standard deviations for the BMI z-score, 0.5 percentage points in the probability of children being overweight or obese, and 0.3 percentage points in the probability of children being obese, respectively. The effects of predicted male employment growth rates on children’s weight status are exhibited in the third row of Table 2. These coefficients have no significant effects. The magnitude of the effects on the male labor market growth rate is also 3 ~ 4 times smaller than the estimates on the female labor market growth rate. In sum, the results in Table 2 highlight that children’s weight is pro-cyclical with respect to female labor market conditions but not with respect to male labor market conditions. This is consistent with studies showing differing effects of male and female labor market conditions on other child outcomes. For example, Page et al. [21] show that improvements in the labor market for women are associated with worse health outcomes for children, while an improvement in labor market conditions for men have small but positive effects on children’s health. Anderberg et al. [25] and Lindo et al. [26] find that male employment is associated with reductions in child maltreatment and female employment is associated with an increase in child maltreatment. Using individual-level employment status data, Phipps, Lethbridge, and Burton [49], Ruhm [30], and Courtemanche [33] also find a significant relationship between a mother’s work hours and children’s weight status but do not find significant effects of changes in father’s work hours on children’s weight status. The insignificant effects of the male labor market in our study are consistent with the results of these studies.

**Table 2:** The effect of predicted employment growth rates on children’s weight status

	(1) BMI Z-score	(2) Overweight/ Obesity	(3) Obesity
Predicted employment growth rates (overall)	0.0005 (0.0020)	0.0010 (0.0016)	0.0023 (0.0002)
Predicted employment growth rates (Female)	0.0124** (0.0039)	0.0045* (0.0022)	0.0029** (0.0012)
Predicted employment growth rates (Male)	− 0.0038 (0.0027)	− 0.0013 (0.0010)	− 0.0006 (0.0007)
N	1,509,740	1,509,740	762,133

Note: Robust standard errors are clustered by county and year. Overweight/obesity in column (2) and obesity in column (3) indicate probability. * p < 0:1, ** p < 0:05, ***p < 0:01

To check whether the impacts are heterogeneous across sub-samples, we estimate eq. (2)–(3) by children’s gender, grade, and race. The effects of the gender-specific predicted employment growth rates for these sub-samples are reported in Table 3. The predicted employment growth rates are not recalculated by sub-sample because data on the industry of employed persons in the 1990 census are not available by race. Since we mainly focus on the effects of the gender-specific growth rates, estimates from the predicted overall employment growth rates are omitted in Table 2 and tables hereafter. Consistent with the results in Table 2, most of the coefficients for predicted male employment growth rates in Table 3 show no significant effects. Predicted female employment growth rates, in contrast, show positive impacts that are significant for certain sub-groups. Results in panel A show stronger effects for girls. Crepinsek and Burstein [50] find that teenage girls with working mothers are more likely to skip the morning meal, which is a risk factor for childhood obesity [51]. The results in panel A correspond to this finding. In terms of heterogeneous impacts by grade shown in panel B, the likelihood of being overweight or obese for 6th–8th-10th grade children is significant. It is not significant for kindergarten-2nd-4th grade children. Arkes [40] also finds significant effects of economic conditions on weight status among older children (15 to 18 years) and Morrissey et al. [35] find that the effect of maternal employment is larger for older children than younger children. Together with the results from these studies, the results in panel B suggest that younger children are less affected by economic conditions compared to older children. It is possible that parental investments in younger children do not change much with economic conditions. For example, for parents, it is possible that the costs of monitoring their children’s dietary choice or physical activity could be lower for younger children since parents typically spend more time with their children when they are younger rather than older. Therefore, if parents consider “parenting” more important when the children are younger rather than older, then they might maintain the investment level in their younger children regardless of the economic conditions. Lastly, we find stronger effects of an increase in the improvement in the female labor market on weight among African Americans. African Americans constitute the largest minority group in Arkansas and, on average, tend to have lower socioeconomic status. Hence, the results may suggest that socioeconomically disadvantaged groups are more vulnerable to the change in female labor market conditions. Chia [29], von Hinke Kessler Scholder, and Miller [31] also found larger maternal employment effects for low socioeconomic status groups. However, our findings are contrary to the results of Anderson et al. [28], Ruhm [30], and Courtemanche et al. [38] who report that the effects of maternal employment on children’s weight are stronger among socioeconomically advantaged households. As Ziol-Guest et al. [52] pointed out, if mothers from higher socioeconomic backgrounds are more capable of managing their children than disadvantaged mothers, the effects of losing time with children can be larger for socioeconomically advantaged groups. On the other hand, the effects can be larger for disadvantaged households if more advantaged households compensate for the negative effect of maternal employment by purchasing healthy inputs for children. Our results support the latter argument. The key takeaway from Table 3 is that the principal finding on the divergence between the effect of male and female employment growth rates reported above holds up across the different subsamples.

**Table 3:** The effect of predicted employment growth rates on children’s weight status: Sub-sample results

	(1)	(2)	(3)
	BMI Z-scores	Overweight/Obesity	Obesity
**Panel A-1: Boys**			
Predicted employment growth rates (Female)	0.0093* (0.0050)	0.0031 (0.0038)	0.0006 (0.0024)
Predicted employment growth rates (Male)	−0.0032 (0.0026)	− 0.0016 (0.0014)	0.0000 (0.0009)
N	776,644	776,644	776,644
**Panel A-2: Girls**			
Predicted employment growth rates (Female)	0.0158*** (0.0044)	0.0061** (0.0017)	0.0052** (0.0017)
Predicted employment growth rates (Male)	−0.0046 (0.0033)	−0.0010 (0.0012)	− 0.0012 (0.0008)
N	733,096	733,096	733,096
**Panel B-1: K-2nd-4th**			
Predicted employment growth rates (Female)	0.0081 (0.0070)	0.0027 (0.0025)	0.0025 (0.0020)
Predicted employment growth rates (Male)	−0.0008 (0.0044)	0.0002 (0.0016)	0.0003 (0.0009)
N	805,775	805,775	805,775
**Panel B-2: 6th–8th-10th**			
Predicted employment growth rates (Female)	0.0116* (0.0058)	0.0058*** (0.0018)	0.0010 (0.0021)
Predicted employment growth rates (Male)	−0.0007 (0.0026)	0.0001 (0.0009)	0.0009 (0.0007)
N	703,965	703,965	703,965
**Panel C-1: White**			
Predicted employment growth rates (Female)	0.0068 (0.0062)	0.0027 (0.0020)	0.0008 (0.0015)
Predicted employment growth rates (Male)	−0.0046 (0.0035)	−0.0022 (0.0014)	− 0.0011 (0.0008)
N	987,911	987,911	987,911
**Panel C-2: African-American**			
Predicted employment growth rates (Female)	0.0165* (0.0084)	0.0057*** (0.0015)	0.0060*** (0.0018)
Predicted employment growth rates (Male)	−0.0006 (0.0028)	0.0007 (0.0013)	0.0008 (0.0004)
N	328,040	328,040	328,040
**Panel C-3: Hispanic**			
Predicted employment growth rates (Female)	0.0265** (0.0096)	0.0072 (0.0050)	−0.0001 (0.0062)
Predicted employment growth rates (Male)	−0.0040 (0.0059)	−0.0004 (0.0033)	0.0004 (0.0030)
N	148,302	148,302	148,302
**Panel C-4: Asian and other**			
Predicted employment growth rates (Female)	0.0376 (0.0209)	0.0155 (0.0119)	0.0026 (0.0092)
Predicted employment growth rates (Male)	−0.0315** (0.0117)	−0.0093 (0.0054)	− 0.0053 (0.0065)
N	45,481	45,481	45,481

Note: Robust standard errors are clustered by county and year Overweight/obesity in column (2) and obesity in column (3) indicate probability. * p < 0:1, ** p < 0:05, ***p < 0:01

We perform several robustness and specification tests to assess the sensitivity of the results in Table 2. First, in addition to individual, county, and time fixed effects, we control for a county-specific linear time trend in the model to account for the parallel existing trends between female labor market participation and increasing overweight and obesity. Next, Lindo et al. [48] point out that the estimated links between economic conditions and health outcomes are sensitive to the level of geographic aggregation and show that more aggregate analysis yields a larger magnitude in the estimated effect because it can capture spillover effects of economic conditions on health outcomes across counties. To account for this in our analysis, we repeat the estimation using larger regional designations from the Arkansas Department of Health, each of which encompasses multiple counties. Specifically, we aggregate our geographic units into five different regions within Arkansas (Central, Northwest, Northeast, Southwest, and Southeast) and estimate eq. (3) using region fixed effects and robust standard errors clustered by region and year. Lastly, to take endogenous migration between counties into account, we estimate samples consisting only of non-movers defined as children whose residences are always observed to be in the same county. Table 4 reports the results, which are broadly consistent with our main results in Table 2.^5^

**Table 4:** Robustness checks of results in Table 2

	(1)	(2)	(3)
	BMI Z-scores	Overweight/ Obesity	Obesity
**Panel A: County specific linear time trend**			
Predicted employment growth rates (Female)	0.0088** (0.0043)	0.0036* (0.0019)	0.0006 (0.0015)
Predicted employment growth rates (Male)	0.0004 (0.0020)	0.0001 (0.0009)	0.0012* (0.0007)
N	1,509,740	1,509,740	1,509,740
**Panel B: Regional predicted employment growth rates**			
Predicted employment growth rates (Female)	0.0138* (0.0058)	0.0055** (0.0020)	0.0023*** (0.0002)
Predicted employment growth rates (Male)	−0.0022 (0.0021)	0.0010 (0.0011)	0.0005 (0.0006)
N	1,509,740	1,509,740	1,509,740
**Panel C: Non-movers**			
Predicted employment growth rates (Female)	0.0132*** (0.0036)	0.0049* (0.0024)	0.0028** (0.0011)
Predicted employment growth rates (Male)	−0.0037 (0.0026)	−0.0010 (0.0010)	− 0.0007 (0.0008)
N	1,298,450	1,298,450	1,298,450

Note: Regional predicted employment growth rates are defined as the average value of the rates of counties in that region. In region level analysis, we use region fixed effect, and robust standard errors are clustered by region and year. Robust standard errors are clustered by individual in other panels. Overweight/obesity in column (2), and obesity in column (3) indicate probability. * *p* < 0.1, ** *p* < 0.05, *** *p* < 0.01

### Mechanisms

To this point, our results can be summarized as follows: an improvement in female labor market conditions is associated with an increase in the BMI z-score and the probability of a child being overweight or obese, while an improvement in male labor market conditions is not significantly associated with children’s weight. To investigate underlying mechanisms for the results in Table 2, following the specification of Cawley and Liu [53] and Abramowitz [54], we estimate eq. (4) using individual-level time use data from the ATUS through OLS:

4 $$ {T}_{ast}={\beta}_0+{\beta}_1{ME}_{ast}+{\beta}_2F{ME}_{ast}+{\beta}_3{X}_{ast}+{\omega}_{ast} $$

where *a* indicates a respondent of the ATUS, *s* indicates state in which the respondent resides, and *t* is the year. *T* indicates time use in minutes for activities that are related to the child’s weight. “Food preparation,” “purchasing prepared food,” “physical activities with children,” and “paid childcare and medical care” are used as activities that could plausibly be related to the child’s weight.^6^ Since the ATUS does not contain information about the county of residence during our study period, the gender-specific predicted employment growth rates, *ME* and *FME*, in eq. (4) are calculated at the state level. In addition, as noted earlier, because the number of respondents from Arkansas in the ATUS is too small to yield meaningful results, we use data from all states in the ATUS. In eq. (4), *X* indicates a vector of control variables for individual characteristics of the respondent. We include binary variables for gender, age, race, educational attainment, marital status, and respondent’s and spouse’s full-time employment status. All regressions are weighted by the ATUS final weights, and robust standard errors are clustered by individual. Since we focus on children’s weight status, samples are restricted to respondents from households with one or more children aged less than 18 years, and to respondents aged between 21 and 64 years old.

The coefficient in column (1) of Table 5 in the first row is for predicted female employment growth rates and shows that a one percentage point increase in predicted female employment growth rates is significantly associated with about 3.5 fewer minutes devoted for preparing food at home each day. The coefficient for purchasing prepared food in column (2) is also significant, but shows a negative sign implying that a one percentage point increase in predicted female employment growth rates is associated with fewer minutes devoted to purchasing prepared food. Recall that we expect the change in female labor market conditions to impact the substitution of time-intensive childcare with market-based childcare. The negative relationship between improvement in female labor market and time for purchasing prepared food does not correspond to this prediction. One possible explanation for the reduction in time for purchasing prepared food is that women are more likely to be involved not only in food preparation but also in food purchasing. In fact, Crepinsek and Burstein [50] who used the Continuing Survey of Food Intakes by Individuals (CSFII) data show that 75% of mothers are the only adult in the household who usually shops for food. In other words, the results can be interpreted that as mothers become more involved in work-related activities, the time spent on preparing and buying food at home is reduced. Hence, improvement in the female labor market could decrease the time spent in food shopping. Cawley and Liu [53] also find that there is a lack of offsetting activities by male partners in response to mother’s employment. This is consistent with an explanation that a decrease in time spent purchasing processed/prepared foods may decrease children’s weight given that foods purchased away from home are known to have higher calories than foods made at home [55]. However, the magnitude of the coefficient for time spent purchasing prepared food is small compared with the decrease in time for food preparation. This may explain why the effects of improved female employment growth rates on BMI z-score and on the probability of being overweight or obese in Table 2 are positive. Note that an alternative explanation for reduced time spent on purchasing food is purchasing food in larger quantities per shopping occasion. If this is the case, then reduced time spent on purchasing food may not necessarily be associated with a decrease in children’s weight. Although we cannot directly test this hypothesis due to data limitations, we think this is another possible explanation for our results given the role of mothers in food-preparation-related work inside the home. We do not find significant effects of improvement in female labor market on time for physical activities with children in column (3) and on time for paid childcare and medical care in column (4). Although it is insignificant, the negative sign of the coefficient in column (4) also may be due to the fact that mothers are mainly responsible for child care. In our sample, females spend about three times more time on paid childcare and medical care than males. Again, given the lack of offsetting activities by male partners, this may explain the result in column (4). In terms of predicted male employment growth rates, we find significant effects for time for purchasing prepared food in column (2), and time for paid childcare and medical care in column (4). In particular, the increase in time for paid childcare and medical care suggests that improvement in the male labor market has a beneficial effect (decrease in weight) on children’s weight through enhanced health investments for children. Moreover, the magnitude of the coefficient for paid childcare and medical care is larger than that of purchasing prepared food, which might have adverse effects on children’s weight. Nevertheless, the magnitudes of both coefficients are less than 1. This explains why the overall effects of improvement in predicted male employment growth rates in Table 2 are negative, although small and insignificant. In sum, the results exhibited in Table 5 indicate that the increase in children’s weight when the female labor market improves is possibly due to a decrease in time for meal preparation in the home.

**Table 5:** The effect of predicted employment growth rates on time for activities related with children’s weight

	(1)	(2)	(3)	(4)
	Food preparation	Purchasing food	Physical activities with children	Paid childcare and medical care
Predicted employment	−3.541**	−0.308***	0.480	−0.153
growth rates (Female)	(1.396)	(0.110)	(1.413)	(0.414)
Predicted employment	0.535	0.188***	−1.373	0.575**
growth rates (Male)	(0.940)	(0.072)	(0.978)	(0.289)
N	32,058	32,058	32,058	32,058

Note: Unit of dependent variables is minutes. Robust standard errors are clustered by individual. * p < 0:1, ** p < 0:05, ***p < 0:01

## Discussion

One of our findings is that a one percentage point increase in the predicted female employment growth rate is associated with a small increase of 0.5 percentage points in the probability of child being overweight or obese. Because earlier studies have reported a positive correlation between maternal employment and children’s weight, we compare the magnitude of our results with those from these other studies. Table 6 contains a summary of the results from several existing studies. We focus on the results for probability of overweight or obesity from the main specification of these studies. In most previous studies, average hours worked per week is used as a measure of maternal employment level. Although the empirical strategies, sample, and study periods differ across the studies, the magnitude of the estimated effects of 10 h more work per week are 1.6 ~ 2.5 percentage points increase in the probability of being overweight, except in von Hinke Kessler Scholder [31] who evaluate the effect of full-time employment status on children in Britain, and by Courtemanche et al. [38] who exploit the age of the youngest child as an instrumental variable for mother’s work hours. These two studies find much larger effects in terms of magnitude (i.e., 5.5 ~ 7.3 percentage points). Although it is not possible to directly compare our results to the findings of other studies given that our employment level measure is at an aggregate level, we can infer that the effect in this study is about one-third or one-fifth of those studies. Courtemanche [33] reports that an additional 10 h of work per week for women increases childhood overweight by 11.1%. Following his procedure, the effect size we report here would translate into 2 ~ 3 h more work per week, which would be associated with an increase in the probability of childhood overweight by 3.1%.^7^ Even though the magnitude of the coefficient is not large, the results of this paper are not trivial in that childhood obesity is influenced by a variety of factors.

**Table 6:** Related research on maternal employment and children’s being overweight

Study	Measure of maternal employment	Geography	Effect on the probability of being overweight
Phipps, Lethbridge, and Burton [49]	Average hours work per week (15 h)	Canada	2.7 percentage points
Chia [29]	average hours work per week (10 h)	Canada	2.5 percentage points
Ruhm [30]	average hours work per week (20 h)	United States	3.0 ~ 4.5 percentage points
Von Hink Kessler Scholder [31]	Full time employment	Britain	5.5 ~ 5.7 percentage points
Courtemanche [33]	average hours work per week (10 h)	United States	1.6 percentage points
Ziol-Guest et al. [52]	average hours work per week (10 h)	United States	1.2 percentage points
Courtemanche, Tchernis, and Zhou [38]	average hours work per week (10 h)	United States	6.6 ~ 8.1 percentage points
This study	Predicted employment growth rates (1 percentage point)	United States (Arkansas)	0.5 percentage points

## Conclusion

In this paper, we found that improvement in the female labor market is associated with an increase in children’s weight status and this may be due to a decrease in time spent preparing meals in the home. The labor force participation rate of women has sharply increased in the last decades. For instance, while the labor force participation rate of men has decreased from 83.4% in 1960 to 69.1% in 2017, the rate for women has increased from 37.8 to 57.0% during same period [56]. Since human capital is one of the essential factors for economic growth, higher female labor participation is encouraged. To the extent that the findings in this study show the unintended negative effects of improvement in female labor markets on children’s health, the policy implications of our paper should be aimed at mitigating the adverse effects of women’s labor participation. For example, because children spend considerable amount of time outside home, efforts to improve school meal quality or the physical activity related built environment may offset the negative effects of maternal employment on children’s well-being. Future research on finding the most effective ways to offset the negative effects of maternal employment on children’s health outcomes would be a worthwhile undertaking.

There are several limitations of this study. First, the results of this study cannot exactly tell us to what extent our results are due to labor market opportunities of females. This is because we cannot observe the individual’s actual behavior related to employment, and economic fluctuation involves changes in other factors. For example, during an economic boom, it is not only employment rates that could increase but also local taxes, which can affect the quality of school meals or other school programs that may influence children’s weight. Even though our measurements for labor market opportunity are exogenous to labor supply, we could not take these factors into account. Moreover, our main results pertain to Arkansas counties, but the analysis was conducted using state-level data from across the county. As mentioned above, this is because there is no information about county of residence and the number of observations from Arkansas is too small to derive meaningful results in the ATUS. We note however that when we restrict the sample to states experiencing high incidence of childhood obesity similar to Arkansas, we still find significant results (the results are available upon request). Hence, we acknowledge that the results for the mechanisms may not reflect the main results on BMI given the differences in populations. Therefore, our results for the mechanisms represent suggestive evidence. Further formal investigation will be required when relevant data are available.

## Data Availability

The data used in this study is housed in the Arkansas Center for Health Improvement (ACHI) in Little Rock, Arkansas. Our use of the Arkansas BMI data is governed by the legislative authority under which the Arkansas Center for Health Improvement (ACHI) has permission to maintain these data (Act 1035 of 2003). Currently, ACHI does not have permission to share data with external researchers. Should someone wish to access these data for replication purposes, they could do so by entering into a data use agreement with ACHI. This would entail a cost to cover ACHI’s expenses of coordinating access and would allow researchers with on-site access to the dataset.

## Abbreviations

BMI: Body Mass Index
ATUS: American Time Use Survey
NLSY: National Longitudinal Survey of Youth
CPS: Current Population Survey
LAUS: Local Area Unemployment Statistics
BLS: Bureau of Labor Statistics
ACS: American Community Survey
NAICS: North American Industry Classification System
ACHI: Arkansas Center for Health Improvement
CDC: Centers for Disease Control and Prevention
OLS: Ordinary Least Squares
CSFII: Continuing Survey of Food Intakes by Individuals

## Footnotes

1 In addition, unemployment rates may not be an indicator that fully reflect the labor market condition in that if the number of people looking for a job increases, the unemployment rate can decrease regardless of the number of people who are actually employed.

2 Thus, the base period industry composition is constructed with more recent data, which could elicit more endogeneity issues. For example, if there was a child health boom in some counties in the late 1990s, this could affect both children’s weight status in 2004 and industry composition in 2000. However, we also acknowledge that composition of employment too far away from study period may be substantially different from the current composition.

3 Note that the predicted employment growth rates for CPS and 1990 Census in table 1 are constructed using counties of Arkansas. However, as explained later, we constructed the predicted employment growth rates for ATUS at the state level.

4 Specifically, 23 vs 50.01 (males vs females) for food preparation, 1.16 vs 1.27 for purchasing prepared food, 7.73 vs 9.18 for physical activities with children, and 0.23 vs 0.82 for paid childcare and medical care.

5 In addition, given the large sample size, we randomly dropped observations and re-estimated the primary specification using smaller samples to check whether the statistical significance of our results is an artifact of the large sample size. We still find significant results using much smaller sample sizes. The results are available from the authors upon request.

6 Examples of food preparation are food and drink preparation, food presentation, and kitchen and food clean-up. Examples of purchasing prepared food are paying the pizza delivery person, buying fast food, and picking up take-out food. Examples of physical activities with children are playing with children (not sports) and playing sports with children. Examples of paid childcare and medical care are hiring a nanny or babysitter, paying for lessons, and giving the child medicine.

7 This result is driven by “[(proportion of children who live with their mothers or another female guardian×change in the probability of overweight for children who live with their mother due to improvement in female labor market opportunity)/proportion of overweight children]”, which is (0.92×0.005)/0.149. 2010 data are used for proportion of children who live with their mothers or another female guard and proportion of overweight children. The first data are from America’s Families and Living Arrangements, and the second data are from Fryar et al. [7].
